# Editorial: Rising stars in nutrition, psychology and brain health: chemosensory signals, nutrition, and eating behavior

**DOI:** 10.3389/fnut.2023.1203576

**Published:** 2023-05-22

**Authors:** Pengfei Han, Julia Y. Q. Low, Minghai Fu

**Affiliations:** ^1^Faculty of Psychology, Southwest University, Chongqing, China; ^2^DINE Lab, School of Science, Royal Melbourne Institute of Technology, Bundoora West Campus, Bundoora, VIC, Australia; ^3^School of Pharmacy, Hainan Medical University, Haikou, China

**Keywords:** chemosensory signals, taste, smell, metabolic diseases, receptors, eating behavior, nutrition, diet-related diseases

The chemosensory system is vital in maintaining homeostasis in humans and animals. However, the complex mechanisms behind the detection, transmission, and modulation of chemosensory cues by homeostasis are not fully understood. Chemosensory processing is postulated to involve a multifaceted flow of events, including nutrient sensing mechanisms in the brain and gut, learning, memory, and reward systems. Therefore, understanding the chemosensory receptor's ability to sense nutrients in response to metabolic changes can inform disease treatment and suggest directions for nutrition wellbeing.

This Research Topic focuses on the burgeoning interdisciplinary field seeking to understand the role of chemosensory perception in nutrition and health. In this special issue, we present a collection of five articles that explore various aspects of sensory nutrition association, including epidemiology, sensory psychophysics, physiology of chemical senses, and the impact of stress, emotion, reward, and mood on chemosensory perception, paving the way for nutritional tools to study chemosensory perceptions and their potentially broad role in human health and diet-related diseases. The commonality between these articles, including those by Tuluhong and Han, Kwon et al., Yeung, Zhang et al., and Briard et al. lies in their exploration of the complex interactions between chemosensory perception, food ingredients, micronutrients, stress, reward, functional sensory cues, and higher-level cognitive processing of food information, all of which contribute to our understanding of nutrition and health. Below are the key highlights from the papers published.

Tuluhong and Han investigated the relationship between chronic stress, food reward, and the perception of food and non-food odors among college students. The results showed that subjective perceived stress levels significantly correlated with the emotional and reward-based eating behaviors. In addition, the daily stressor exposure was associated with decreased liking for low-calorie sweet foods.

Kwon et al. explored the impact of coffee intake on the sympathetic response to acetylcholine and the sudomotor function. This study revealed that high concentrations of caffeine intake could stimulate higher activation in axon reflexes and sweat responses, implying that caffeine may activate nicotinergic axon reflexes and muscarinergic acetylcholine signaling and supporting its further implications in the activation of the cholinergic and sudomotor functions as a clinical supplement.

Yeung conducted a meta-analysis of fMRI studies examining the neural correlates of food labels (nutritional label, food brand, food nature) as stimuli. Results showed that food labels were generally processed in brain regions related to internal mentation of self-consciousness and nutritional evaluation, but the correlates of food brand and food nature labels were inconsistent across findings, highlighting the need for further research.

Zhang et al. investigated the cross-sectional association between vitamin K intake and depressive symptoms in United States adults using a large dataset from the National Health and Nutrition Examination Survey (NHANES) 2013–2018. The study reported an inverse linear relationship between vitamin K intake and depressive symptoms in a sample of 11,687 adults, after adjustments of potential confounding variables. Such findings provide initial evidence that increased vitamin K intake may be associated with a reduced likelihood of depressive symptoms.

Using functional magnetic resonance imaging (fMRI), Briard et al. examined the effects of supplementation with a sensory functional ingredient (flavor) on the behavioral and brain responses of juvenile pigs to acute stress. The study demonstrated that previous exposure to the functional ingredient modulated the brain activation in the brain areas involved in food pleasure and motivation, while alleviating the brain responses to acute stress in animals such as pigs. This study offers insights into the role of chemosensory signals (flavor) on stress adaptation.

The research presented in this special issue emphasizes the significant progress made in understanding the role of sensory nutrition association in health and wellbeing management. These studies offer valuable insights into the complex interactions between chemosensory perception and environmental factors, including findings from brain imaging in animals. Moreover, the articles underscore the importance of continued investigation in this field to develop targeted or personalized sensory-nutritional interventions that could potentially be used to manage diet-related diseases and wellbeing. By addressing various aspects of this multifaceted field, the articles collectively contribute to the growing body of knowledge on sensory nutrition association, highlighting its potential connections with other aspects of human health and wellbeing. As such, the diverse Research Topics presented in this issue underscore the importance of a comprehensive understanding of chemosensory perception in human health and disease management, paving the way for continued investigation and critical analysis.

Collectively, the articles included in this Research Topic demonstrate the findings from various techniques on the relationship between chemosensory perception and wellbeing. We sincerely hope you find this collection of articles interesting and inspiring. These studies contribute to the expanding knowledge base on sensory nutrition association and emphasize its potential impact on human health and wellbeing. By continuing to explore this fascinating area of research, we can develop a deeper understanding of the role chemosensory perception plays in nutrition, psychology, and brain health, ultimately leading to improved health outcomes and wellbeing management strategies.

## Author contributions

All authors wrote the first draft, made critical revision, and approved the final version of the manuscript.

